# Deciphering the clinical and genetic spectrum of early-onset inborn errors of immunity in a Brazilian pediatric cohort

**DOI:** 10.3389/fimmu.2026.1839133

**Published:** 2026-06-24

**Authors:** Luiza de Mattos, Mayara Mayer Alves, Nickolas Piller Wegbecher, Ana Paula Diniz Iwamura, Irma Cecilia Douglas Paes Barreto, Marina Hideko Kinoshita Assahide, Flavia A. T. M. Guimarães, Patricia Faddul, Gisele Kuntze, Herberto Jose Chong-Neto, Rafaela Wagner, Victor Horacio de Souza Costa Júnior, Saloe Bispo, Laire Schidlowski, Carolina Prando

**Affiliations:** 1 Instituto de Pesquisa Pelé Pequeno Príncipe, Curitiba, Brazil; 2 Faculdades Pequeno Príncipe, Curitiba, Brazil; 3 Hospital Pequeno Príncipe, Curitiba, Brazil; 4 Centro Universitário do Estado do Pará, Belém, Brazil; 5 Hospital Materno Infantil de Brasília, Brasília, Brazil; 6 Clínica Les Grands Petits, Londrina, Brazil; 7 Hospital de Clínicas da Universidade Federal do Paraná, Curitiba, Brazil

**Keywords:** genetic diagnosis, inborn errors of immunity, next generation sequencing, rare disease, South America

## Abstract

Inborn errors of immunity are a heterogeneous group of rare genetic disorders associated with susceptibility to infections, autoimmunity, allergy, and malignancy. Owing to marked clinical variability, phenotypic overlap, and limited access to specialized diagnostic resources, their diagnosis remains challenging. To investigate the diagnostic contribution of whole-exome sequencing in this setting, we studied 100 Brazilian children up to 4 years of age with early-onset manifestations suggestive of inborn errors of immunity. Exome findings were classified as conclusive diagnoses, suggestive diagnoses, or alternative genetic diagnoses, according to variant pathogenicity and phenotype correlation. The cohort included children from 48 municipalities across Brazil, with a mean age of 16.7 ± 12.9 months at recruitment. A conclusive molecular diagnosis was established in 17% of cases. In this group, 21 disease-causing variants were identified, one-third of which were novel. Thirteen individuals had suggestive genetic findings, whereas five were diagnosed with conditions outside the inborn errors of immunity classification; in this latter group, two-thirds of the variants were novel. The most frequent category among conclusive diagnoses was combined immunodeficiencies with associated features or syndromes. Investigation of probands also led to the identification of 21 additional affected family members. Importantly, the molecular findings had a direct effect on clinical care, including the indication for hematopoietic stem cell transplantation in >50% of index cases with conclusive diagnoses, as well as implications for targeted treatment, genetic counseling, and reproductive planning. These findings demonstrate that whole-exome sequencing is a valuable diagnostic approach in children with early and complex presentations suggestive of inborn errors of immunity, while also contributing to genomic knowledge in an underrepresented population.

## 1 Introduction

Inborn errors of immunity (IEIs) are a group of rare diseases associated with >500 genes that cause increased susceptibility to infections, autoimmunity, allergies, and tumors in affected individuals (1). IEIs are considered rare diseases, although recent studies show an increasing prevalence, with estimates reaching 1/1100 (2, 3). Although IEIs are being identified more frequently, diagnosing this class of diseases remains challenging. Similar to other rare diseases, knowledge of such conditions among the general population and healthcare professionals is limited, and access to reference centers and laboratories capable of performing special tests is restricted (4, 5).

As >7000 different rare diseases exist, with estimates reaching up to 10 000, divergence in the time required to diagnose these conditions is expected. In general, studies have indicated that the average duration of the diagnosis odyssey for rare diseases is 5 years (5, 6). In the context of IEIs, time-to-diagnosis also shows considerable variability. Cohort characteristics, such as age, clinical suspicion, and clinical presentation contribute to this time-to-diagnosis (7–9). Moreover, the broad spectrum of clinical manifestations associated with IEIs further influences the duration of diagnostic delay, as some conditions present with mild symptoms, whereas others manifest with life-threatening features. In addition, some IEIs have very similar phenotypes, making it challenging to define the condition being evaluated accurately (1). Not only do IEIs overlap phenotypically with each other but they also overlap with diseases not considered immune disorders, which can lead to delayed diagnosis or misdiagnosis and, consequently, incorrect treatment. Such delays increase the risk of complications and reduce the quality of life and life expectancy of affected individuals (10). The literature indicates that early treatment improves prognosis and reduces risks such as organ failure and systemic infection. These findings highlight the importance of diagnosing IEIs in the first months of life to reduce the risk of future complications (11, 12). Therefore, accurate identification of the genetic cause is crucial for selecting appropriate treatment, planning clinical follow-up, and improving the development of new therapeutic options.

The last few decades have been marked by an increasing number of genes associated with monogenic diseases due to the widespread use of next-generation sequencing (NGS). According to Bamshad et al. (13), between 2010 and 2017, 87% of genes associated with Mendelian genetic disorders were identified using NGS techniques. The identification of genes associated with IEIs has followed the same trend. Numerous new IEI-associated genes are described in each publication by the International Union of Immunological Societies (IUIS) expert committee. Over less than 15 years, publications from the IUIS committee have documented a marked increase in the number of described IEIs, rising from 191 conditions in 2011 to 559 in 2024 (1, 14, 15). Limited access to genetic testing in developing countries, such as Brazil, represents an important challenge for molecular diagnosis (45). In addition, a large gap in Brazilian genomic data exists due to the lack of large-scale studies in this population. This limitation complicates variant interpretation and analysis, given that Brazil has one of the largest admixed populations worldwide (16). This study investigated an exclusively pediatric cohort of 100 individuals, and to our knowledge, this study represents the largest population with this profile investigated for IEI in Brazil to date.

In the present study, we report the results of exome sequencing in a Brazilian cohort of 100 children up to 4 years of age who presented with phenotypes compatible with IEI.

## 2 Materials and methods

### 2.1 Patients

The present study used a prospective recruitment approach with retrospective chart review. A total of 100 Brazilian patients up to 4 years of age at the time of recruitment were included. Participants were prospectively selected based on the presence of history of recurrent infection as defined by the Jeffrey Modell Foundation 10 warning signs (46). After integrated analysis of the index cases, including clinical data, laboratory findings, and WES, an additional 152 family members were included for genetic segregation analysis and investigation of symptomatic relatives.

### 2.2 Clinical and laboratory data collection

Clinical and laboratory data were collected through structured review of electronic medical records and standardized interviews with family members using a predefined clinical form. Clinical manifestations were categorized according to the affected systems, including the central nervous system, upper airways, lower airways, skin, cardiovascular system, urinary tract, gastrointestinal tract, musculoskeletal system, ocular system and systemic infections. Data on IEI-related noninfectious manifestations, as defined by the Jeffrey Modell 10 warning signs, were also collected. Previous laboratory results were evaluated when available, including complete blood count, immunophenotyping for T-cell subpopulations or primary immunodeficiency orientation tube (PIDOT), immunoglobulin levels, complement protein levels, T-cell receptor excision circle (TREC) and (kappa-deleting recombination excision circle (KREC) values, and dihydrorhodamine (DHR) oxidation test. When appropriate, age-adjusted reference ranges were applied to laboratory test interpretation. The number of hospital admissions for each participant was recorded, along with the length of stay, categorized as 1–15, 15–30, 30–60, 60–90, and >90 d. Hospital admissions and clinical manifestations were considered from the first admission until the cutoff date in May 2025.

### 2.3 Whole exome sequencing

Peripheral blood was collected from participants, and DNA was extracted using the ReliaPrep™ Blood gDNA Miniprep System (Promega, Madison, WI, USA), following the manufacturer’s instructions. DNA quantification was performed via UV-Vis spectrophotometry using a NanoDrop instrument (Thermo Fisher Scientific, Waltham, MA, USA).

WES was performed on an Illumina NovaSeq 6000 platform (Illumina, San Diego, CA, USA) using a capture library covering all exons and exon-intron boundaries of the human genome, targeting a mean coverage depth of approximately 100×. Exome enrichment was performed using the xGen Exome Research Panel v2 (Integrated DNA Technologies, Coralville, IA, USA). Bioinformatic processing was performed using a Snakemake-based pipeline following Genome Analysis Toolkit (GATK) best practices. Reads were quality-filtered using fastp (minimum Phred score, 20; minimum length, 20 bp) and aligned to the GRCh38/hg38 reference genome using BWA-MEM. Aligned reads were sorted, duplicates were marked using Picard, and base quality score recalibration was performed using GATK with standard known variant databases. Single-nucleotide variants (SNVs) and insertions/deletions (indels) were called using GATK HaplotypeCaller within Agilent SureSelect target regions, applying a minimum confidence threshold of 30.

#### 2.3.1 Copy number variant analysis using ExomeDepth

Copy number variant (CNV) analysis was performed using the ExomeDepth R package (47). Baseline samples included equal numbers of female and male individuals. For each sample, an optimized reference set was generated by selecting samples with highly correlated coverage profiles. CNVs were inferred from normalized read depth using a beta-binomial model and classified as deletions or duplications based on copy number estimates. Detected CNVs were annotated and integrated with variant interpretation analyses, considering the presence of IEI-associated genes. The detected CNVs consistent with the clinical presentation and the expected mode of inheritance were not experimentally validated using methods such as MLPA or qPCR. To ensure the quality and reliability of the CNVs identified, a coverage assessment of the genes involved was performed across the entire cohort (n=100). Information regarding the quality standards can be found in **Supplementary Material 1**. CNV interpretation was conducted in accordance with the joint ACMG/ClinGen consensus recommendations for the interpretation and reporting of constitutional CNV (17).

#### 2.3.2 Variant calling and interpretation

The Wannovar tool (https://wannovar.wglab.org) and Franklyn by Genoox (https://franklin.genoox.com) were used for WES analysis. Analyses were performed separately for each participant, prioritizing variants based on clinical and laboratory findings and the following criteria: a panel of 508 IEI-associated genes (1); allele frequency <0.01 in the 1000 Genomes and Genome Aggregation Database (gnomAD); pathogenicity scores from prediction tools such as SIFT (https://siftdna.org/), Polyphen (http://genetics.bwh.harvard.edu/pph2), and AlphaMissense (https://alphamissense.hegelab.org); and exclusion of synonymous variants. In cases in which no variants associated with the clinical presentation were identified, the analysis was expanded to non-IEI-associated genes according to the 2025 IUIS classification. Based on variant prioritization, information was collected from databases such as ClinVar, Online Mendelian Inheritance in Man (OMIM), and scientific literature to accumulate evidence. Identified variants were evaluated according to the American College of Medical Genetics and Genomics and the Association for Molecular Pathology (ACMG/AMP) criteria (17) to determine pathogenicity. The analysis workflow can be found in **Supplementary Material 2**.

### 2.4 Sanger sequencing

For family segregation analysis and diagnosis of symptomatic relatives, Sanger sequencing was performed. Genomic DNA was amplified via PCR using a DNA polymerase amplification kit (Thermo Fisher Scientific, Waltham, MA, USA), with oligonucleotides designed according to the location of the variant under investigation. Oligonucleotides were designed using the Primer-BLAST tool. PCR products were labeled with BigDye v3.1 reagent (Thermo Fisher Scientific, Waltham, MA, USA) according to the manufacturer’s instructions, using a reaction containing 20 ng of PCR product. Samples were sequenced using a Genetic Analyser 3500xL instrument (Thermo Fisher Scientific, Waltham, MA, USA). Electropherograms and base sequences were generated using the Sequencing Analysis v5.4 software (Thermo Fisher Scientific, Waltham, MA, USA) and analyzed using FinchTV (Geospiza, Inc., Seattle, WA, USA).

### 2.5 Statistical analysis

Categorical variables were analyzed using both descriptive and inferential statistics. Variables were initially presented as absolute (n) and relative (%) frequencies, stratified according to three classification categories: conclusive, suggestive, and alternative genetic diagnoses.

The association between classification categories and sex and age variables was assessed using the chi-square test of independence. Given the small cell sizes in the contingency table, statistical significance was estimated using the Monte Carlo permutation method, which provides a more robust approximation when asymptotic assumptions are not fully met.

For the variable family history suggestive of an inborn error of immunity, the k proportions chi-square test was applied to evaluate differences in distribution among the three groups. Wherever necessary, multiple comparisons between proportions were performed using the Marascuilo test. A significance level of 0.05 was used for all analyses.

### 2.6 Ethics

The study was approved by Hospital Pequeno Príncipe Research Ethics Committee (CAAE: 57914522.9.0000.0097 - approval number 5.579.288/2022), in accordance with Resolution 466/12 of the National Health Council. Data collection was initiated after approval was obtained. Written informed consent was obtained from the minor’s legal guardian for the publication of data included in this article. The electronic medical records of participants were accessed to collect clinical and laboratory data. The recorded data were anonymized and transferred to a spreadsheet.

## 3 Results

### 3.1 Patient profile

Among the 100 Brazilian participants investigated for suspected IEI, 62% were male, whereas 38% were female. The study sample comprised individuals originating from 48 municipalities across Brazil. All participants were recruited at a mean age of 16.7 ± 12.9 months. The consanguinity rate in the cohort was 4%, and 7% had a family history suggestive of IEI, although without genetic confirmation.

During the study period, 81 individuals were hospitalized at least once. Among them, 48 required admission to the intensive care unit (ICU), representing 59.25% of hospitalized participants. Overall, participants experienced a total of 174 hospitalizations, of which 59.77% lasted up to 15 days. The most frequent hospitalization pattern was single admission, representing 51.85% (n = 42) of hospitalized individuals. Whereas, 17.28% (n = 14) participants were admitted twice, and 9.87% (n = 8) three times. Conversely, 19% of participants had no history of hospitalization despite reporting recurrent infections.

### 3.2 WES results

We classified patients into groups according to exome analysis results. All variants and/or CNVs consistent with the participant’s clinical presentation and expected inheritance pattern were classified according to the ACMG/AMP or ACMG/ClinGen criteria. The conclusive genetic diagnosis group included cases with pathogenic or likely pathogenic variants in IEI-associated genes (1). The suggestive genetic diagnosis group included (a) cases with variants of uncertain significance (VUS) or (b) variants previously described as pathogenic or likely pathogenic but with incomplete penetrance or (c) risk alleles (e.g. *TNFRSF13B, MEFV, G6PD*). The group of alternative genetic diagnoses comprised genetic diagnoses not associated with IEI, despite similar phenotypes. Clinical characteristics of the three groups are presented in **Table 1**, and the identified variants are summarized in **Table 2**.

**Table 1:** Main clinical characteristics across conclusive, suggestive and alternative diagnoses groups.

Conclusive diagnoses	
IUIS IEI classification	Main symptoms
Combined immunodeficiencies with associated features or syndromes	Syndromic features; Dermatitis; Lower respiratory tract infection; Upper respiratory tract infection; Hypogammaglobulinemia; High levels of IgE; Developmental delay; Gastrointestinal infection; Low TRECs.
Immune dysregulation diseases	Syndromic features; Cytopenias; Hepatomegaly; Vaccine adverse reaction; Lower respiratory tract infection; Mucocutaneous lesions; Macrophage activation syndrome.
Immunodeficiencies affecting cellular and humoral immunity	Lower respiratory tract infection; Sepsis; Urinary tract infection; Gastrointestinal manifestations; Low TRECs; Lymphopenia T; Lymphopenia B.
Congenital defects of phagocytes	Abscess; Lower respiratory tract infection; Sepsis; Gastrointestinal manifestations; Candidiasis; Mycobacterial infection.
Phenocopies of IEI	Cutaneous manifestations; Monocytosis; Cytopenias.
Defects of innate immunity	Mycobacterial infection.
Suggestive diagnoses	
Predominantly antibody deficiencies	Lower respiratory tract infection; Upper respiratory tract infection; Gastrointestinal manifestations; Hypogammaglobulinemia; Cutaneous manifestations; Sepsis.
Congenital defects of phagocyte	Cutaneous manifestations; Lower respiratory tract infection; Mucocutaneous lesions; Urinary tract infection; Hypogammaglobulinemia.
Autoinflammatory disorders	Persistent fevers; Cutaneous manifestations; Meningitis; Musculoskeletal manifestations; Hypogammaglobulinemia; Sepsis.
Alternative genetic diagnoses	
Alternative genetic diagnoses	Lower respiratory tract infection; Sepsis; Persistent fever; Urinary tract infection; Cutaneous manifestations; Upper respiratory tract infection; Hypogammaglobulinemia.

**Table 2:** Details of molecular findings in participants.

Gene	Transcript	Variant	Classification criteria*	Classification
*ADA*	*NM_000022.4*	c.320T>C p.(Leu107Pro)	PM1, PM2, PP2, PP3, PP5	Pathogenic
*ADA*	*NM_000022.4*	c.845G>A p.(Arg282Gln)	PM2, PM3, PP2, PP3, PP5	Pathogenic
*ADA*	*NM_000022.4*	44651118-44652220del	2C	Likely pathogenic
*ADA2*	*NM_001282225.2*	c.140G>C p.(Gly47Ala)	PM1, PM2, PM5, PP2, PP3	Pathogenic
*ATM*	*NM_000051.4*	108121502-108407182del	2A	Pathogenic
*ATM*	*NM_000051.4*	108365082-108365508del	2E, 4L	Pathogenic
*CARD11*	*NM_032415.7*	c.119C>A p.(Ala40Asp)	PS3, PM1, PM2	Likely pathogenic
*CCDC39*	*NM_181426.2*	c.357 + 1G>C	PVS1, PM2, PP5	Pathogenic
*CCDC39*	*NM_181426.2*	180651402-180652521del	2E	Likely pathogenic
*CNTNAP1*	*NM_003632.3*	c.3741dupA p.(Ser1248fs)	PVS1, PM2, PP4	Pathogenic
*CYBB*	*NM_000397.4*	c.769T>A p.(Cys257Ser)	PS3, PM2, PM5, PP3	Pathogenic
*CYBB*	*NM_000397.4*	c.1499A>T p.(Asp500Val)	PM1, PM2, PM5	Likely pathogenic
*CYBB*	*NM_000397.4*	c.1354G>T p.(Glu452Ter)	PVS1, PM2	Likely pathogenic
*G6PD*	*NM_001360016.2*	c.202G>A p.(Val68Met)	PS3, PM1, PP5, BA1	VUS
*G6PD*	*NM_001360016.2*	c.376A>G p.(Asn126Asp)	PS3, PM1, PP5, BA1	VUS
*GLB1*	*NM_000404.4*	c.1577dup p.(Trp527LeufsTer5)	PVS1, PM2, PP5	Pathogenic
*IL10RA*	*NM_001558.4*	c.742G>A p.(Gly248Arg)	PS3, PM2, PP3	Likely pathogenic
*IL12RB1*	*NM_005535.3*	c.409 + 1G>A	PVS1, PM2, PP4	Pathogenic
*IL2RG*	*NM_000206.3*	c.206A>G p.(Tyr69Cys)	PM1, PM2, PP3, PP4	Likely pathogenic
*KMT2D*	*NM_003482.4*	c.10615C>G p.(Arg3539Gly)	PM1, PM2, PM5, PP2	Likely pathogenic
*KRAS*	*NM_004985.5*	c.38G>A p.(Gly13Asp)	PS3, PM1, PM2, PM5, PP3, PP5	Pathogenic
*MEFV*	*NM_000243.3*	c.2084A>G p.(Lys695Arg)	PM1, PM2, PM5, PP5	Likely pathogenic
*MEFV*	*NM_000243.3*	c.2230G>T p.(Ala744Ser)	PM1, PM2, PM3, PP5	Likely pathogenic
*NIPBL*	*NM_133433.4*	c.2683_2684dup p.(Lys896LeufsTer34)	PVS1, PM2	Likely pathogenic
*RAB27A*	*NM_183235.3*	c.550C>T p.(Arg184Ter)	PVS1, PM2, PP5	Pathogenic
*RAB27A*	*NM_183235.3*	c.467 + 1G>C	PVS1, PS3, PM2	Pathogenic
*RAB27A*	*NM_183235.3*	55196354-55205705del	2D	Likely pathogenic
*RAB27A*	*NM_183235.3*	55223890-55234956del	2C	Likely pathogenic
*RAB27A*	*NM_183235.3*	55234783-55234956del	2C	Likely pathogenic
*STAT3*	*NM_139276.3*	c.1144C>T p.(Arg382Trp)	PM1, PM2, PM5, PP2, PP3, PP5	Pathogenic
*TNFRSF13B*	*NM_012452.3*	c.118T>C p.(Trp40Arg)	PM2, PP3	VUS
*TNFRSF13B*	*NM_012452.3*	c.260T>A p.(Ile87Asn)	PS3, PS4, PM2, PP5	Pathogenic
*TNFRSF13B*	*NM_012452.3*	c.542C>A p.(Ala181Glu)	PS3, PS4, PM2, PM5, PP5	Pathogenic
*TP63*	*NM_003722.5*	c.T1672C p.(Cys558Arg)	PM1, PM2, PM5, PP2, PP3	Likely pathogenic
*WAS*	*NM_000377.3*	c.777 + 1G>T	PVS1, PM2	Likely pathogenic

*Classification criteria followed ACMG/AMP criteria for single nucleotide and indels and ACMG/ClinGen for copy number variation.

VUS, Variant of uncertain significance.

Regarding family history suggestive of IEI, we detected a higher frequency in the conclusive group (35%), followed by the alternative diagnoses group (20%), whereas no cases were observed in the suggestive group. The analysis indicated a trend toward a difference between groups, although this did not reach statistical significance (*p* = 0.0618).

Regarding age of onset, most individuals in the conclusive group presented their first symptoms before 1 month of age (47%). In the suggestive group, the 2–6-month age group predominated (54%) and in the alternative diagnoses group, we observed the highest frequency in the <1-month age group (80%). A comparison of the categories revealed no statistically significant differences in age distribution (*p* = 0.2061).

### 3.3 Diagnostic yield

The rate of conclusive genetic diagnoses was 17% (n = 17). Diagnoses were based on 21 variants considered causative of the phenotype. Among these, 16 were SNVs and five were CNVs. Of the SNVs, 11 were missense, three were splice-site variants, and two were nonsense variants. We identified five variants in heterozygous states with autosomal dominant inheritance. Four diagnoses were based on eight variants in compound heterozygosity, and three diagnoses were based on homozygous variants associated with autosomal recessive conditions. Additionally, five cases involved hemizygous variants. Among the variants identified in the conclusive group, 57.1% (12/21) were not reported in population databases and were considered novel.

In the suggestive diagnoses group, 13 individuals carried variants that may contribute to the phenotype. Although the variants are considered of uncertain significance or risk alleles, and thus does not provide a definitive diagnosis, their identification may be informative. This does not imply that individuals in this group will necessarily manifest the phenotype associated with the condition. All variants were previously reported SNVs, with 9 being heterozygous and 4 hemizygous.

We identified five individuals with alternative genetic diagnoses. In this group, six variants were considered causative and classified as pathogenic or likely pathogenic, with inheritance patterns consistent with the phenotype. Five variants were SNVs, whereas one was a CNV; two were homozygous, one was compound heterozygous, and two were heterozygous. Among these six variants, four (66.6%) were novel.

### 3.4 Profile of conclusive diagnoses

The mean age of participants with a conclusive diagnosis was 18.76 months at recruitment and all participants presented their first symptoms within the first 2 years of life. The mean time-to-diagnosis was 14.18 ± 12.16 months.

The most frequent immunodeficiency category among conclusive diagnoses was “combined immunodeficiencies with syndromic features” (five cases: *CARD11*; *STAT3; WAS*; *ATM*; *KMT2D*). Other frequent categories included “diseases of immune dysregulation” (*IL10RA*; *RAB27A*), “immunodeficiencies affecting cellular and humoral immunity” (*IL2RG*; *ADA*), and “congenital defects of phagocytes” (*CYBB*), each with three cases. We also identified single cases in the categories “defects in intrinsic and innate immunity” (*IL12RB1*), “phenocopies of IEIs” (*KRAS*), and “autoinflammatory diseases” (*ADA2*).

#### 3.4.1 Familial segregation

Based on proband results, we established 21 additional diagnoses in family members; notably, nine were IEI diagnoses (*CARD11*, *ADA2*, *CYBB*) and 12 were carriers of X-linked conditions (*CYBB*; *IL2RG*, *WAS*). We also identified other 13 heterozygous carriers of variants associated with autosomal recessive conditions (*IL12RB1; IL10RA*; *RAB27A*; *ADA*).

#### 3.4.2 Clinical effect

After diagnosis, we referred six participants for hematopoietic stem cell transplantation (HSCT). Referred cases included: interleukin-10 receptor α deficiency, severe combined immunodeficiency due to common gamma chain deficiency, Griscelli syndrome, Wiskott-Aldrich syndrome, chronic granulomatous disease, and severe combined immunodeficiency due to adenosine deaminase deficiency. The remaining participants with confirmed diagnoses and indications for HSCT are awaiting treatment and are receiving prophylactic therapy, immunobiological treatment, or follow-up with an immunology team. Genetic counseling was provided to families with confirmed diagnoses.

## 4 Discussion

The present study investigated a large, exclusively pediatric Brazilian cohort with early-onset symptoms suggestive of IEI, achieving a diagnostic yield of 17%, identifying a substantial proportion of novel variants, and demonstrating a significant effect on clinical management and family-based diagnosis. These findings highlight the relevance of systematic genomic investigation in children with early and complex clinical presentations.

The growing number of genes associated with IEI, as reported by the IUIS, has increased diagnostic complexity due to significant phenotypic overlap between conditions and phenotypic expansion associated with the same genes. In this context, NGS, including WES and WGS, has been highly useful for disease elucidation, contributing to diagnostic accuracy, improved clinical management, and more precise prognosis (1, 18).

The diagnostic yield observed in the present cohort (17%) is consistent with previously reported rates in studies using NGS for suspected IEIs, which generally range from 10% to 46% depending on cohort characteristics and study design (9, 19–22). Although some studies have reported higher rates of conclusive genetic diagnoses, these are often associated with preselected populations with strong clinical suspicion or confirmed IEIs, or cohorts with high consanguinity rates. These findings suggest that even in real-world tertiary care settings with broad inclusion criteria, WES provides a consistent and clinically meaningful diagnostic yield.

The present cohort comprised patients referred with heterogeneous and often complex clinical presentations in a setting with low consanguinity (4%), which is associated with a moderate diagnostic rate. Differences in diagnostic yield across studies may be explained by population characteristics, including consanguinity rates, sequencing approaches, inclusion criteria, and age distribution. Studies reporting higher yields often included preselected cohorts or populations with high consanguinity. For example, Erman & Çipe (20) achieved a genetic diagnosis in 63% of patients with prior clinical suspicion of IEI, while Simon et al. (21) reported a 70% diagnostic rate in a cohort with 62% consanguinity. The complexity of cases included in the present study is reflected in the diversity of conditions affecting multiple organ systems, some of which initially mimicked IEIs. This finding is demonstrated by the diagnoses made in the group of alternative genetic diagnoses.

An important contribution of this study lies in the characterization of a large, exclusively pediatric cohort from Brazil with very early-onset manifestations. Most individuals presented symptoms within the first year of life, with a substantial proportion in the first months, reflecting enrichment for severe phenotypes. This profile differs from many previously published cohorts that include broader age ranges or a substantial proportion of adults.

Additionally, participants were recruited from high-complexity tertiary centers, likely contributing to the concentration of severe and multisystemic cases. While the enrichment for complex and severe phenotypes in this cohort might increase the likelihood of identifying monogenic disorders through genomic approaches, it may also limit generalizability of the findings, as individuals with milder phenotypes identifiable through standard immunological evaluation would be more likely to be detected and managed outside this setting. This may also explain the identification of conditions not classified as IEIs but presenting with overlapping phenotypes, reinforcing the diagnostic challenge. Conversely, individuals with milder phenotypes or conditions diagnosable through standard immunological evaluation may have been managed outside this setting.

Notably, investigation of probands enabled the diagnosis of 21 additional family members, emphasizing the broader effect of genomic diagnosis beyond the index case and reinforcing its importance for genetic counseling and clinical management.

Among conclusive diagnoses, 21 variants were identified, of which 57.1% (12/21) were not reported in population databases. In the group of alternative genetic diagnoses, 66.6% (4/6) of variants were novel. Brazil has one of the largest admixed populations worldwide, which confers high genomic diversity. However, it remains underrepresented in major genomic databases. In 2025, Nunes et al. investigated the genomes of 2723 Brazilians and identified >8 million previously unreported variants, including >36–000 potentially deleterious variants (16). These findings reflect a gap in genomic knowledge due to limited large-scale studies in Brazilian populations, complicating variant interpretation and highlighting the importance of expanding genomic research in underrepresented populations. Notably, all novel variants identified in the present cohort (57.1% in the conclusive and 66.6% in the group of alternative genetic diagnoses) were also absent from the population analyzed by Nunes et al., further emphasizing this gap. These observations support the inclusion of diverse populations in global genomic databases to improve diagnostic accuracy.

The distribution of IEI categories among conclusive diagnoses in the present cohort was characterized by a predominance of “combined immunodeficiencies with associated features or syndromes” (n = 5), followed by “immune dysregulation diseases,” “immunodeficiencies affecting cellular and humoral immunity”, and “congenital defects of phagocytes” (three cases each). The least prevalent categories were “phenocopies of IEI,” “defects of innate immunity, “ and “autoinflammatory diseases,” with one case each. The studies by Zhu et al. and Tengsujaritkul et al. reported results very similar in early age Chinese and Thai population when compared to our study (23, 24), with “combined immunodeficiencies with associated features or syndromes”, representing 60% and 38.8% of genetic diagnoses, respectively. In Malaysia, Ripen et al. studied children with a mean age of 1 year, and even though the “combined immunodeficiencies with associated features or syndromes” category was not prevalent, they found “autoinflammatory diseases”, “immune dysregulation diseases”, and “innate immunity defects,” each representing 21.42% of total genetic diagnoses (25), which were also representative groups in our cohort.

Primary antibody deficiencies (PAD), which represent the most prevalent category within the largest cohort of individuals diagnosed with inborn errors of immunity (26), was not observed in our population. This can be explained by the average age of our patients, who have not yet reached the minimum age required, for example, to confirm a diagnosis of CVID, the primary diagnosis in the Kindle study. Furthermore, Kindle reports that among the categories of IEIs, PAD had the lowest proportion of genetic diagnoses (12.4%), even though it involved the largest number of patients. Considering age, even within pediatric cohorts, the prevalence of primary antibody deficiency (PAD) diagnoses increases with rising mean patient age. This can be exemplified in the cohort described by Ferreira (27), which evaluated Brazilian patients with a mean age of 10 years and reported a 53.84% rate of genetically confirmed PAD. In adult populations, such as the studies published by Okano et al. and Elsink et al., the class “predominantly antibody defects” accounted for a large proportion of genetic diagnoses, respectively 45, 9% e 20% (9, 28).

Thus, the age restriction for inclusion in this study may account for the lack of diagnoses within the PADs. In addition, most of our participants were recruited from a high-complexity pediatric hospital, which predominantly manages more severe cases and patients whose conditions could not be resolved using standard diagnostic approaches available at lower-complexity health services, such as immunoglobulin quantification or immunophenotyping. Therefore, some patients with antibody deficiency may have received a diagnosis without being reported for genetic testing.

In the present cohort, the time to diagnosis among patients with conclusive diagnosis was relatively short compared with that reported in previous studies, which often describe prolonged diagnostic odysseys in IEIs (7–9). This difference may be influenced by the exclusively pediatric nature of our cohort, the early onset of symptoms, the inclusion of patients evaluated in tertiary centers, and early access to genomic testing. The Immunodeficiency Foundation (IDF) gathered information from 977 individuals with IEI and reported an average delay of 25 years between the first symptom and diagnosis, with a mean age of 59 years (29). The age profile of participants in the IDF study reflects a period with more limited access to genetic testing, which may explain the longer diagnostic time compared with that observed in the present study. The Romanian cohort published by Pantea et al., is the most comparable to the present study, as both are exclusively pediatric and have a mean age below 5 years (7). However, the longer time-to-diagnosis in that study may be explained by a wider age range among participants, including individuals up to 18 years of age.

The relatively short diagnostic time observed in the present study reflects the inclusion criteria of a maximum age of 4 years. Notably, all patients with conclusive diagnoses experienced symptom onset before 2 years of age. This suggests that, in most cases, symptoms appeared very early and highlights the importance of early recognition of clinical manifestations. Early suspicion can lead to more prompt genetic testing and thus minimize diagnostic delays.

Developed countries with better access to healthcare are better equipped to provide specialized centers and professionals capable of identifying rare diseases, thus promoting faster diagnosis (30). However, population-specific factors can also contribute to variation in diagnostic time, such as consanguinity rates, which increase the occurrence of autosomal recessive diseases (30, 31). Advances in artificial intelligence tools for early detection of IEIs, expansion of neonatal screening, and establishment of specialized centers, are expected to shorten the diagnostic odyssey (32, 33).

The suggestive diagnoses group illustrates the complexity of variant interpretation in IEIs, particularly in the context of incomplete penetrance, variable expressivity, or uncertain clinical significance. Variants in genes such as *TNFRSF13B* and *MEFV* exemplify this challenge, as monoallelic alterations may contribute to disease susceptibility without being sufficient to establish a definitive diagnosis (9, 34–39). This was observed in our cohort, as variants identified in probands were also detected in clinically unaffected parents in cases of suspicion of familial mediterranean fever and TACI deficiency.

The identification of alternative genetic diagnoses not classified as IEIs but presenting with overlapping clinical features highlights the phenotypic complexity of this patient population. In addition to the clinical manifestations, laboratory abnormalities, including severe lymphopenia, contributed to raising the suspicion of IEI. As WES encompasses genes beyond the described clinical picture, secondary or incidental findings are possible (40, 41). This occurred in five confirmed diagnoses in the studied cohort. These data reinforce the importance of performing exome sequencing when investigating individuals with complex clinical presentations, as gene panel testing for IEIs would not reach such diagnoses. Furthermore, although not currently included in the classification of IEIs, two of these diagnoses involve candidate genes for IEIs (*CCDC39* and *CNTNAP1*) (42) and represent cases that could contribute to defining the role of these genes in immune system functions.

Genetic diagnosis had a direct effect on clinical management in the present cohort. The genetics results were particularly important for cases that presented atypical or mild symptoms, despite their impact on the patient’s quality of life. In 64.7% (n=11) of 17 cases with a conclusive diagnosis, genetic testing guided the management approach. Among them, 10 had the recommendation for HTSC, and 1 for intravenous immunoglobulin replacement therapy.

The literature demonstrates a growing trend on expanding the recommendations for HSCT in patients with IEIs (48). The integration of genetic testing with clinical aspects enhances the precision and robustness of these recommendations. More than half of the index cases with conclusive diagnosis (58.82%) had indications for HSCT, whereas others, including patients from the three groups (conclusive, suggestive, alternative), benefited from immunoglobulin replacement or antimicrobial prophylaxis. These findings are consistent with previous studies demonstrating that genomic diagnosis can significantly modify clinical management, including treatment decisions and preventive strategies. Simon et al. reported a change in clinical management in 55% of 74 participants with conclusive results after exome analysis (21). Upon evaluation of 278 families from 22 countries, Stray-Pedersen et al. achieved a comparable molecular diagnosis in 40% of cases, which led to changes in clinical management, including a recommendation for HSCT for 55% of cases (43). In addition to individual patient care, the identification of affected relatives in the present study highlights the broader clinical and familial effect of genomic investigation, enabling early diagnosis, genetic counseling, and informed reproductive planning.

In the present study, WES enabled early diagnosis in several cases, many of which would remain unresolved without genetic analysis. Recent advancements have allowed the emergence of new sequencing methodologies with reduced cost (44). The present findings reinforce the importance of implementing such testing on a larger scale, especially among individuals who present risk factors for genetic diseases, such as a positive family history or very early-onset of symptoms. In addition, the increasing number of genomic sequences contributes to the construction of more representative genetic databases, particularly for populations with poorly characterized and highly admixed genetic backgrounds.

The results obtained corroborate previously published studies under similar conditions, highlighting the benefit of using WES for the diagnosis of IEIs. Despite this, >60% of participants included in the present study remain without a definitive diagnosis, suggesting the possibility of another underlying condition, an atypical phenotype, technical limitations of WES (such as incomplete coverage, limited detection of structural variants and deep intronic variants), or even an IEI that has not yet been described. WGS and periodic reanalysis of exome data may help overcome these limitations and reveal new diagnoses as gene–disease associations continue to be discovered.

In conclusion, the present study conducted a genetic investigation for IEIs in a Brazilian pediatric cohort, and demonstrated that WES is an effective diagnostic tool in children with suspected early-onset IEIs, providing clinically actionable results and enabling timely therapeutic interventions. The characterization of a large Brazilian pediatric cohort contributes to the expansion of genomic knowledge in underrepresented populations and reinforces the importance of integrating genomic approaches into routine clinical practice for rare immune disorders.

## Data Availability

The datasets generated and analyzed for this study are available on request from the corresponding author due to privacy.
