# Supplementary material for: Deciphering the clinical and genetic spectrum of early-onset inborn errors of immunity in a Brazilian pediatric cohort
Source: Front Immunol. 2026 Jun 24;17:1839133. doi: 10.3389/fimmu.2026.1839133 (PMC13341498; doi:10.3389/fimmu.2026.1839133)

Supplementary material 1

Gene-level coverage metrics for CNV analysis.

Gene	Number of exons analyzed per gene	Mean sequencing depth	Median sequencing depth	Minimum exon depth	% of exons with high-quality coverage	% of exons with intermediate coverage	% of exons with low coverage	% of exons with depth ≥20×	% of exons with depth ≥30×
ADA	97	74,64	50	0	62,89	0	37,11	86,6	62,89
ATM	152	44,95	36,5	0	57,24	13,82	28,95	83,55	64,47
CCDC39	44	38,77	32	0	50	11,36	38,64	84,09	56,82
RAB27A	37	29,27	0	0	24,32	8,11	67,57	40,54	32,43

CNV calls were inferred by calculating the likelihood of copy number states and summarized using Bayes factors.
To ensure robustness, the following criteria were applied:
- Statistical support: CNV calls were required to present a strong Bayes factor (BF > 10), indicating significant deviation from the diploid model.
- Consistency across targets: We considered only events supported by consistent read-depth deviation across one or more contiguous exons.
- Coverage constraints: CNV calls were restricted to regions with sufficient sequencing depth.
- median exon coverage ≥ 20–30x
- ≥80–90% of samples with coverage ≥20xReference set optimization: The reference set was selected using the select.reference.set function to maximize correlation between test and reference profiles, reducing technical noise and false positives.

Stringent segmentation: A transition probability of 10^{-4} was used to limit excessive segmentation and reduce spurious CNV calls.

Supplementary material 2
Workflow of genomic analysis.

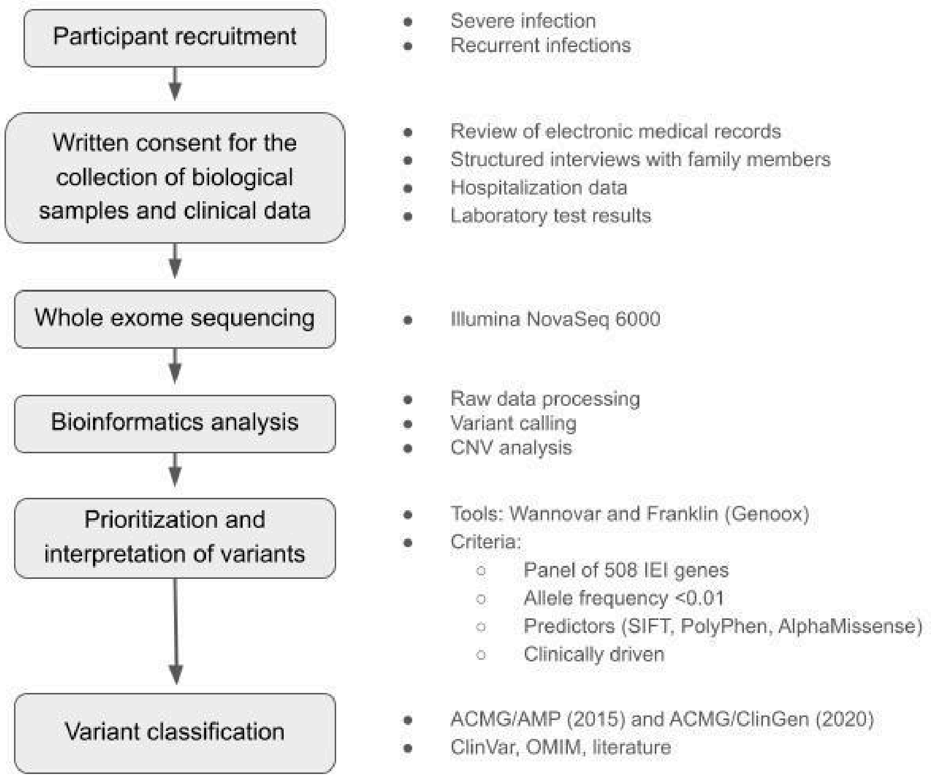

Supplement: Supplementary file 1 [file DataSheet1.pdf]
